# Multidimensional Prognostic Index (MPI) in elderly patients with acute myocardial infarction

**DOI:** 10.1007/s40520-020-01718-6

**Published:** 2020-10-01

**Authors:** Valeria Cammalleri, Michela Bonanni, Francesca Maria Bueti, Andrea Matteucci, Lisa Cammalleri, Giuseppe Stifano, Saverio Muscoli, Francesco Romeo

**Affiliations:** 1 Department of Cardiovascular Disease, Tor Vergata University, Via Montpellier 1, 00133 Rome, Italy; 2 Department of OrthoGeriatrics, Rehabilitation and Stabilization, Galliera Hospital, Genova, Italy

**Keywords:** Frailty, Geriatric assessment, Acute coronary syndrome, Acute myocardial infarction

## Abstract

**Background:**

Management of elderly patients with acute myocardial infarction (AMI) is challenging due to lack of knowledge about the link between fragility, outcomes and interventional procedures.

**Aims:**

The aim of this study was to establish the prognostic role of the Multidimensional Prognostic Index (MPI) in elderly with AMI.

**Methods:**

A total of 241 patients ≥ 65 years old with AMI were continuously enrolled in this prospective study and divided into three groups according to the MPI score. The primary endpoint was 30-day mortality. Secondary endpoints were 6-month mortality and rate of adverse events.

**Results:**

In-hospital overall mortality rate was higher in MPI-3 (*p* = 0.009). Patients of MPI-3 had a significantly higher mortality rate regarding the primary endpoint with 30-day survival of 78.9%, compared to 97.4% and 97.2%, in MPI-1, MPI-2 (*p* < 0.001), respectively. The survival rate progressively decreased in the three MPI classes of risk with a 6-month survival of 96.5%, 96.3%, 73.7% in groups MPI-1, MPI-2, and MPI-3 (*p* < 0.001). Longer length of in-hospital stay was observed in MPI-3 group. In-hospital complications were more frequent in higher MPI score.

**Discussion:**

Our findings are in agreement with the results of other studies that evaluated the risk of in-hospital complications and mortality in older patients. In our “real-world” population of elderly hospitalized for AMI we observed poorer outcomes in patients belonged to higher MPI groups.

**Conclusions:**

In the setting of AMI, MPI may be very useful in the daily clinical practice to manage older patients and predict the risk of in-hospital and follow-up complications.

## Introduction

In Western countries, the number of elderly patients with complex needs for healthcare is large and growing as a result of demographic and epidemiological causes. The most common diagnostic category for these patients is cardiovascular disease, and the vast majority of patients admitted to cardiology units are currently elderly [1, 2], including those suffering from the acute coronary syndrome.

Hospitalization in older patients for an acute event represents a stressful condition that can lead to severe complications, death, prolonged length of stay and functional decline more frequently than younger patients. Poorer outcomes are likely because the elderly have more comorbidities, increased risk of bleeding and are less likely to receive reperfusion or medical standard therapy compared with young [1–5]. There is a great heterogeneity among elderly, therefore, identifying those patients at high risk of mortality is crucial for decision making. Interactions between normal biological aging processes in the cardiovascular system, age-related pathology, sequelae of heart disease, comorbidity, frailty and polypharmacy can influence the benefit-risk ratio of all interventions. A multidimensional approach, taking into account pathological, psychological and environmental factors is pivotal to assess life expectancy of older patients [6]. The Multidimensional Prognostic Index (MPI) based on comprehensive geriatric assessment, has been shown to predict mortality in hospitalized older patients for an acute disease or a relapse of a chronic disease [7, 8]. However, a clear prognostic role of MPI in older patients admitted to a cardiology department for acute coronary syndrome has not been yet explored.

The aim of our study is to establish the prognostic role of the MPI in older patients hospitalized for acute myocardial infarction (AMI), who underwent a percutaneous coronary intervention (PCI).

## Materials and methods

### Patient population

In this study we prospectively enrolled 241 patients with AMI treated in the cardiology department of “Policlinico Tor Vergata” of Rome with PCI from June 2018 to October 2019. Inclusion criteria were: (1) age ≥ 65 years; (2) diagnosis of ST-segment elevation myocardial infarction (STEMI) or non-ST segment elevation myocardial infarction (NSTEMI) (3) invasive treatment with PCI; (4) ability to provide an informed consent to participate in the study; (5) acquisition of the complete comprehensive geriatric assessment (CGA) during hospitalization.

Our study population was divided into three groups according to the MPI: low risk (MPI-1, *n* = 114), moderate risk (MPI-2, *n* = 108), and severe risk of mortality (MPI-3, *n* = 19).

All patients have been managed according to international guidelines and provided written informed consent [3–5].

### Data collection

At baseline, the following parameters were collected by interview and clinical evaluation: age, gender, clinical and medication history, anthropometric data, cardiovascular risk factors and current pathologies as obstructive pulmonary disease, congestive heart failure, renal impairment, cerebrovascular disease, peripheral vascular disease, malignant disease, anaemia, and dementia. Electrocardiograms were obtained according to routine practice. A standard transthoracic echocardiography was performed at the moment of hospital admission and discharge, recording left ventricle ejection fraction (LVEF), valve abnormalities and possible mechanical complications of myocardial infarction. Laboratory tests including haemoglobin, creatinine, leucocytes, creatine kinase, creatine kinase myocardial band, myoglobin and high-sensitivity troponin I were obtained according to routine practice. The duration of in-hospital stay and adverse events were reported as follows: death, cardiogenic shock, acute pulmonary oedema, implantation of a temporary or permanent pacemaker, use of mechanical circulatory support, acute stent thrombosis, respiratory infections, ventricular fibrillation, sustained ventricular tachycardia, non-sustained ventricular tachycardia, new onset of atrial fibrillation/atrial flutter (AF) and atrioventricular blocks.

### Multidimensional Prognostic Index

A standard comprehensive geriatric assessment (CGA) was performed during a planned specific consultation to calculate the MPI by evaluating eight domains with 63 items as follows: functional status by Activities of Daily Living (ADL) and Instrumental Activities of Daily Living (IALD), cognitive status by Short Portable Mental Status Questionnaire (SPMSQ), nutritional status by Mini Nutritional Assessment (MNA), risk of developing pressure scores by Exton-Smith Scale (ESS), comorbidities by the Cumulative Illness Rating Scale (CIRS), the number of drugs at admission, and co-habitation status. The final MPI score was obtained by summing values of each domain and then dividing them by 8. The result identifies three grades of MPI mortality risk: MPI-1 (≤ 0.33) indicates low risk, MPI-2 (0.34–0.66) moderate risk and MPI-3 (≥ 0.66) high risk. Time required for collecting data for the CGA was about 20 min. Details regarding all the parts of the MPI were detailed somewhere else [7, 8].

### Follow-up and endpoint

A systematic follow-up was performed in all patients. Vital status and all causes of mortality at 30-day and 6-month were assessed by contacting the patients. In addition, we recorded the following adverse events: cardiovascular death, all-causes of hospitalization, re-AMI, stroke or transient ischemic attack, bleedings, device implantation (pacemaker or implantable cardioverter-defibrillator).

The primary endpoint was 30-day mortality of all causes. Secondary endpoints were 6-month all causes mortality and rate of adverse events.

### Statistical analysis

Descriptive statistics were expressed as means ± standard deviations (SD) or medians and interquartile ranges (IQR) for continuous variables. The ANOVA test was used for normally distributed variables and Kruskal–Wallis test for non-normally distributed variables. Categorical variables were presented as absolute number and percentages and were compared with the Chi-square test. Kaplan–Meier survival curves were obtained across MPI risk classes for the different endpoints, and differences between the groups were evaluated with the log-rank test. A two-sided *p* value < 0.05 was considered of statistical significance. Statistical analyses were performed using the Statistical Package for Social Sciences, version 26 (SPSS, Chicago, IL).

## Results

### Study population

A total of 241 patients with a mean MPI score of 0.38 ± 0.16 were consecutively enrolled. According to the MPI score, 114 (47.3%) patients belonged to the MPI-1 group, 108 (44.8%) to MPI-2 group and 19 (7.9%) patients to MPI-3. A greater presence of NSTEMI was observed in more fragile patients (36% vs 45.4% vs 63.2%; *p* = 0.058), while a higher number of STEMI was detected in the MPI-1 and MPI-2 group (64% vs 54.6% vs 36.8; *p* = 0.058). Baseline, demographic and clinical features of MPI groups are summarized in Table 1. As expected, the mean age was significantly different between groups (72.10 ± 5.34 vs 77.44 ± 7.02 vs 82.47 ± 7.43 years old; *p* < 0.0001; respectively). A considerable percentages of patients had conventional risk factors of coronary artery diseases. Specifically, MPI-2 and MPI-3 groups had a higher prevalence of hypertension compared to MPI-1 patients (76.3% vs 89.8% vs 94.7%, *p* = 0.010). In addition, we found a high prevalence of diabetes in higher MPI score groups (27.2% vs 42.6% vs 42.1%, *p* = 0.045), while the majority of active smokers belonged to MPI-1 group (63.1% vs 54.6% vs 21.1%, *p* < 0.001). We also observed a high prevalence of chronic renal failure (10.5% vs 24.1% vs 47.4%, *p* < 0.001) and anaemia in MPI-3 patients (14.9% vs 32.4% vs 68.4%, *p* < 0.001). MPI-3 group had a higher peak of leucocytes (median 11.65 vs 11.23 vs 13.1 thousand/μL, *p* = 0.025). A significant difference was also observed for haemoglobin values at the moment of hospital admission and lower peak of haemoglobin during hospitalization (median 13.9 vs 13.1 vs 11.7 g/dl, *p* < 0.001; 12.7 vs 11.5 vs 10.1 g/dl *p* < 0.001, respectively). Moreover, patients in MPI-3 group showed higher rate of dementia (2.6% vs 25% vs 52.6%, *p* < 0,001). Although the LVEF was reduced in all three study groups, subjects in the MPI-3 showed particularly low values, compared to less frail patients (44.44 ± 9.82 vs 40.97 ± 10.12 vs 37.11 ± 11.46%, *p* = 0.003). Furthermore, MPI-3 patients showed multiple valvulopathies greater or equal to moderate grade. Particularly, they had higher prevalence of mitral insufficiency (75.4% vs 75.9% vs 84.2%; *p* < 0.001), aortic insufficiency (25.5% vs 41.7% vs 73.7%; *p* = 0.001) and tricuspid insufficiency (61.4% vs 65.7% vs 78.9%; *p* < 0.001). Additionally, the systolic pulmonary artery pressure was higher in MPI-3 patients (29.61 ± 13.26; 30.9 ± 11.7; 37.89 ± 15.51 mmHg; *p* = 0.034).

**Table 1:** Baseline characteristics of patients according to MPI

	MPI-1 *n* = 114	MPI-2 *n* = 108	MPI-3 *n* = 19	*p*
**General characteristics**				
Age, years ± SD	72.10 ± 5.34	77.44 ± 7.02	82.47 ± 7.43	< 0.001*
Male, *n* (%)	91 (79.8)	74 (68.5)	9 (47.4)	0.007
Hypertension, *n* (%)	87 (76.3)	97 (89.8)	18 (94.7)	0.010*
Dyslipidaemia, *n* (%)	89 (78.1)	89 (82.4)	17 (89.5)	0.437
Diabetes, *n* (%)	31 (27.2)	46 (42.6)	8 (42.1)	0.045*
Current smoker, *n* (%)	72 (63.1)	59 (54.6)	4 (21.1)	< 0.001*
Previous AMI, *n* (%)	20 (17.5)	42 (38.8)	5 (26.3)	0.007*
History of CVA, *n* (%)	2 (1.8)	14 (13)	5 (26.3)	< 0.001*
Peripheral vascular disease, *n* (%)	3 (2.6)	11 (10.2)	3 (15.8)	0.027*
COPD, *n* (%)	6 (5.3)	11 (10.2)	3 (15.8)	0.193
Malignant disease, *n* (%)	20 (17.5)	19 (17.6)	5 (26.3)	0.436
Chronic renal failure, *n* (%)	12 (10.5)	26 (24.1)	9 (47.4)	< 0.001*
Anaemia, *n* (%)	17 (14.9)	35 (32.4)	13 (68.4)	< 0.001*
Dementia, *n* (%)	3 (2.6)	27 (25)	10 (52.6)	< 0.001*
**Laboratory tests**				
Baseline creatinine (mg/dl), median (IQR)	0.97 (0.28–3.59)	1.05 (1.08–1.28)	1.12 (0.96–1.57)	0.032*
Creatinine peak (mg/dl), median (IQR)	1.16 (1.23–1.64)	1.31 (1.38–1.71)	1.43 (1.36–2.26)	0.275
Discharge creatinine (mg/dl), median (IQR)	1.11 (1.10–1.42)	1.15 (1.16–1.42)	1.15 (1.06–1.76)	0.729
Baseline haemoglobin (g/dl), median (IQR)	13.9 (13.66–14.26)	13.1 (12.4–13..3)	11.7 (10.33–12.67)	< 0.001*
Minimum haemoglobin value (g/dl), median (IQR)	12.7 (12.21–12.94)	11.5 (10.99–11.84)	10.1 (9.24–11.21)	< 0.001*
Discharge haemoglobin(g/dl), median (IQR)	13.5 (12.15–15.66)	12.1 (11.7–12.48)	11.2 (10.22–11.83)	0.061
Leucocytes peak (thousand/μL), median (RIQ)	11.65 (11.53–13.44)	11.23 (11.28–12.91)	13.1 (10.12–21.71)	0.025*
**Echocardiography**				
LVEF, % ± SD	44.44 ± 9.82	40.97 ± 10.12	37.11 ± 11.46	0.003*
Mitral insufficiency, *n *(%)	86 (75.4)	82 (75.9)	16 (84.2)	< 0.001*
Aortic stenosis, *n* (%)	6 (5.3)	16 (14.9)	2 (10.6)	0.026*
Aortic insufficiency, *n* (%)	29 (25.5)	45 (41.7)	14 (73.7)	0.001*
Tricuspid insufficiency, *n* (%)	70 (61.4)	71 (65.7)	15 (78.9)	< 0.001*
sPAP ± DS	29.61 ± 13.26	30.9 ± 11.7	37.89 ± 15.51	0.034*
**MPI-ITEMS**				
ADL, *n* ± SD	5.37 ± 0.50	4.56 ± 1.07	2.00 ± 1.10	< 0.001*
IADL, *n* ± SD	5.44 ± 1.60	3.79 ± 1.57	1.89 ± 0.99	< 0.001*
ESS, *n* ± SD	17.58 ± 1.41	15.05 ± 2.13	12.00 ± 1.86	< 0.001*
SPMSQ, *n* ± SD	1.11 ± 1	3.12 ± 2.41	5.37 ± 2.34	< 0.001*
CIRS, *n* ± SD	5.61 ± 1.72	7.25 ± 1.97	8.53 ± 1.74	< 0.001*
MNA, *n* ± SD	22.05 ± 2.53	18.37 ± 2.72	14.39 ± 2.56	< 0.001*
Total score, *n* ± SD	0.24 ± 0.06	0.47 ± 0.08	0.73 ± 0.04	< 0.001*

*A 2-sided p value <0.05 is considered of statistical significance

*AMI* acute myocardial infarction, *CVA* cerebrovascular accident, *COPD* chronic obstructive pulmonary disease, *LVEF* left ventricular ejection fraction, *sPAP* systolic pulmonary arterial pressure, *ADL* activities of daily living, *IADL* instrumental-ADL, *ESS* Exton-Smith Scale, *SPMSQ* Short Portable Mental Status Questionnaire, *CIRS* Cumulative Index Rating Scale, *MNA* Mini Nutritional Assessment

### In-hospital management and outcomes

All patients underwent successfully PCI with stent implantation. Among patients with STEMI, the majority underwent primary PCI (94.2%), whereas 5.8% underwent rescue PCI. Interestingly, a statistically longer length of in-hospital stay was observed in MPI-3 group (median 6 vs 7 vs 9 days, *p* = 0.035). However, there was no difference between groups in intensive-care length (Table 2). Cardiac biomarker levels were similar between the groups. Patients with severe MPI score had more intra-hospital complications. Specifically, cardiogenic shock and pulmonary oedema occurred mainly in MPI-2 and MPI-3 groups (0.9% vs 7.4% vs 5.3%, *p* = 0.048; 7.9% vs 13% vs 31.6%, *p* = 0.012; respectively). Furthermore, we observed that new onset of AF and need of permanent pacemaker were more frequent in higher MPI scores (9.6% vs 25.9% vs 36.8%, *p* = 0.001; 4.4% vs 10.2% vs 26.3%, *p* = 0.006). In addition, individuals of MPI-3 group seemed to develop more rate of in-hospital pneumonia and consequently more use of antibiotic therapy (11.4% vs 26.9% vs 42.1%, *p* = 0.001; 21.1% vs 38.9% vs 52.6%, *p* = 0.002, respectively). According to the clinical and echocardiographic status, the use of diuretics therapy was 100% among MPI-3 group, compared with 75.9% in MPI-2 and 54,4% of MPI-1 (*p* < 0.001). The administration of transdermal and oral nitrates was significantly higher in MPI-3 group (7.9% vs 13% vs 31.6%, *p* = 0.012). In addition, the use of anticoagulanttherapy in MPI-3 subjects was greater (58%) compared with those in MP-2 (25%) and MPI-1 (10%) groups (*p* < 0.001). The in-hospital overall mortality rate was 2.1%. Respectively 0% (*n* = 0), 2.8% (*n* = 3) and 10.5% (*n* = 2) died in MPI-1, MPI-2 and MPI-3 for cardiac causes (*p* = 0.009).

**Table 2:** Intra-hospital management and outcomes according to MPI

	MPI-1 *n* = 114	MPI-2 *n* = 108	MPI-3 *n* = 19	*p*
**Clinical presentation**				
STEMI, *n* (%)	73 (64)	59 (54.6)	7 (36.8)	0.058
NSTEMI, *n* (%)	41 (36)	49 (45.4)	12 (63.2)	0.058
**In-hospital stay**				
Length of in-hospital stay (days), median (IQR)	6 (5–9)	7 (6–11)	9 (5–18)	0.035*
Length of intensive-care stay (days), median (IQR)	4 (3–5)	4 (3–5)	3 (2–5)	0.659
**Laboratory tests**				
TnI-hs (ng/l), median (IQR)	24,026.6 (45,231.0–74,755.6)	22,728.5 (50,926.9–92,527.6)	15,846.6 (23,925–173,674)	0.985
CK (UI/L), median (IQR)	649.5 (1093.6–1788.0)	566.5 (991.1–1789.4)	550 (399.3–2299.9)	0.718
CK-MB (ng/ml), median (IQR)	76.45 (106.43–166.11)	59.6 (99.24–159.09)	48.3 (40.66–215.53)	0.829
Myoglobin (ng/ml), median (IQR)	326 (822.0–1744.8)	458.5 (958.7–1886.0)	477 (574.12–2448.1)	0.235
**Procedural data**				
P-PCI, *n* (%)	70 (95.9)	55 (93.2)	6 (85.7)	0.492
Rescue-PCI, *n* (%)	3 (4.1)	4 (6.8)	1 (14.3)	0.492
Atrioventricular block, *n* (%)	4 (3.5)	10 (9.3)	0 (0.0)	0.099
Temporary pacemaker, *n* (%)	3 (2.6)	6 (5.6)	0 (0.0)	0.347
IABP, *n* (%)	2 (1.8)	6 (5.6)	1 (5.3)	0.307
**In-hospital complications**				
Death, *n* (%)	0 (0.0)	3 (2.8)	2 (10.5)	0.009*
Cardiogenic shock, *n* (%)	1 (0.9)	8 (7.4)	1 (5.3)	0.048*
Acute pulmonary oedema, *n* (%)	9 (7.9)	14 (13)	6 (31.6)	0.012*
NSVT, *n* (%)	71 (62.3)	75 (69.4)	13 (68.4)	0.516
VF, *n* (%)	9 (7.9)	3 (2.8)	1 (5.3)	0.241
Atrial fibrillation/flutter, *n* (%)	11 (9.6)	28 (25.9)	7 (36.8)	0.001*
Definitive pacemaker, *n* (%)	5 (4.4)	11 (10.2)	5 (26.3)	0.006*
Pneumonia, *n* (%)	13 (11.4)	29 (26.9)	8 (42.1)	0.001*
Heart rupture, *n* (%)	0 (0.0)	3 (2.8)	2 (10.5)	0.009*
Intrastent thrombosis	2 (1.8)	1 (0.9)	0 (0.0)	0.752
**Pharmacological therapy**				
Diuretics, *n* (%)	62 (54.4)	82 (75.9)	19 (100)	< 0.001*
Cardioaspirin, *n* (%)	114 (100)	107 (99.1)	18 (94.7)	0.064
Clopidogrel, *n* (%)	42 (36.8)	65 (60.2)	16 (84.2)	< 0.001*
Others P2Y12 inhibitors, *n* (%)	74 (64.9)	38 (35.2)	2 (10.5)	< 0.001*
Nitrates, *n* (%)	9 (7.9)	14 (13)	6 (31.6)	0.012*
Oral anticoagulant therapy, *n* (%)	12 (10.5)	28 (25.9)	11 (57.9)	< 0.001*
Antibiotic therapy, *n* (%)	24 (21.1)	42 (38.9)	10 (52.6)	0.002*

*A 2-sided p value <0.05 is considered of statistical significance

*STEMI* ST-segment elevation myocardial infarction, *NSTEMI* non-ST segment elevation myocardial infarction, *CK* creatine kinase, *CK-MB* creatine kinase myocardial band, *TnI-hs* high-sensitivity troponin I, *PCI* percutaneous coronary intervention, *PPCI* primary percutaneous coronary intervention, *IABP* intra-aortic balloon pump, *NSVT* non sustained ventricular tachycardia, *VF* ventricular fibrillation

### Follow-up and endpoint

**Fig. 1:**
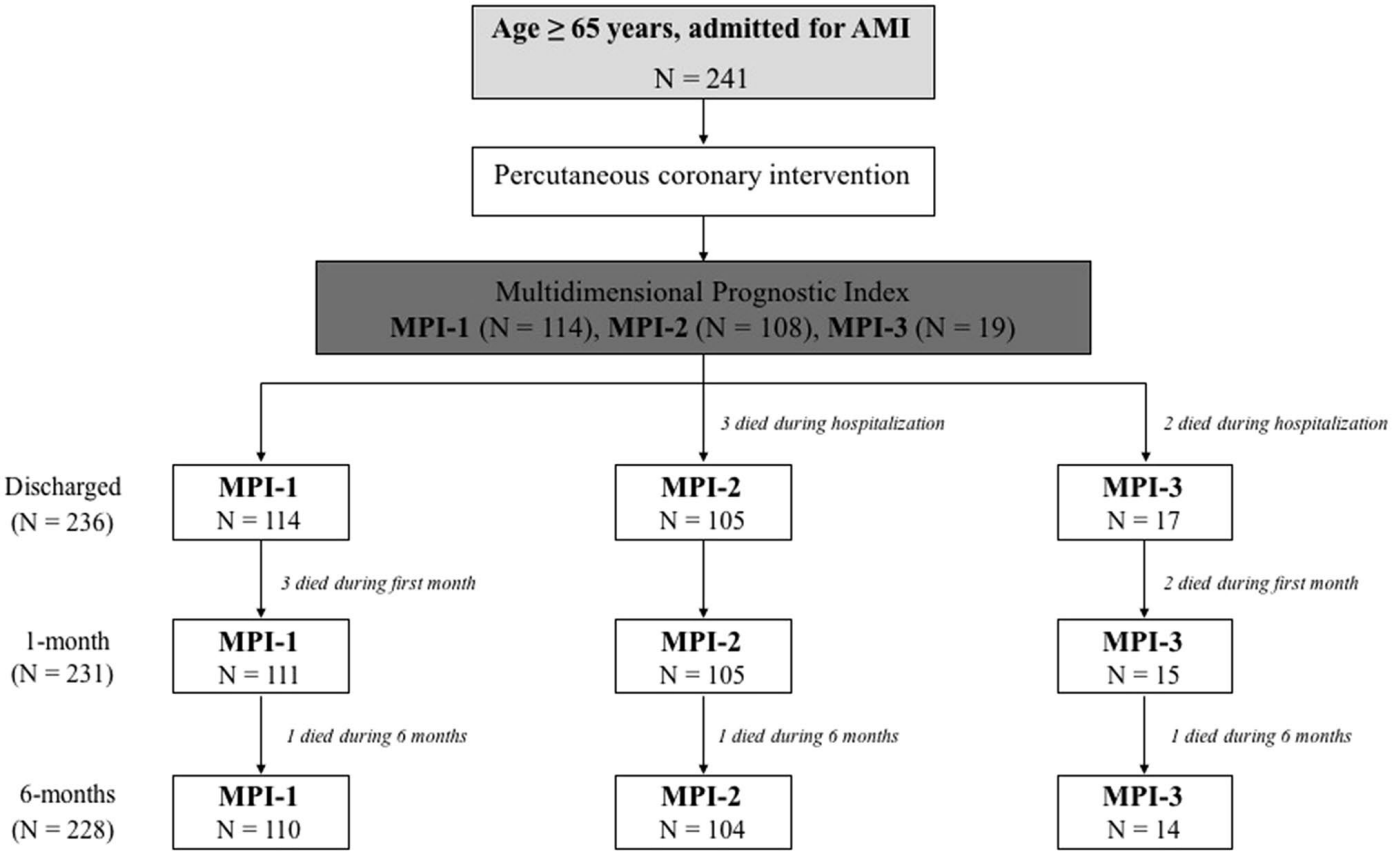
Flow chart illustrating patients included in the study and their distribution according to the Multidimensional Prognostic Index. *AMI* acute myocardial infarction, *MPI* Multidimensional Prognostic Index

No patient was lost to follow-up (Fig. 1). During the follow-up, the rate of adverse events resulted similarly distributed, except for bleedings and stent thrombosis (Table 3). Particularly at 1-month follow-up, MPI-3 group had significant higher bleeding rate compared with lower MPI scores (17.5% vs 27.6% vs 47.1%, *p* = 0.016). In this regard, we noticed that within the MPI-2 and MPI-3 groups, about 60% and 84% were on clopidogrel therapy (*p* < 0.001), in contrast to the MPI-1 group, where 65% of patients were preferably treated with other P2Y12 platelet receptor inhibitors (*p* < 0.001), especially ticagrelor. The rate of stent thrombosis at 30-day was significantly higher in MPI-3 (0% vs 0% vs 5.9%; *p* = 0.02). One patient belonging to MPI-3 group developed haemorrhagic stroke at 6-month follow-up (*p* < 0.001) (Table 3).

**Table 3:** Follow-up and endpoints according to MPI

	MPI-1	MPI-2	MPI-3	*p*
**30-day follow-up**^#^				
Bleedings, *n* (%)	20 (17.5)	29 (27.6)	8 (47.1)	0.016*
Re-AMI, *n* (%)	3 (2.6)	2 (1.9)	2 (11.8)	0.081
Stent thrombosis, *n* (%)	0 (0.0)	0 (0.0)	1 (5.9)	0.02*
Device implantation, *n* (%)	1 (0.9)	3 (2.9)	0 (0.0)	0.612
All causes hospitalization, *n* (%)	17 (14.9)	25 (23.8)	5 (29.4)	0.153
Stroke or TIA, *n* (%)	0 (0.0)	1 (1.0)	0 (0.0)	0.534
**6-month follow-up**^§^				
Bleedings, *n* (%)	37 (33.0)	42 (40)	9 (60)	0.109
Re-AMI, *n* (%)	5 (4.5)	10 (9.5)	2 (13.3)	0.235
Stent thrombosis	0 (0.0)	2 (1.9)	1 (6.7)	0.077
Device implantation, *n* (%)	7 (6.3)	11 (10.5)	0 (0.0)	0.601
All causes hospitalization, *n* (%)	33 (29.5)	44 (42.0)	6 (40 0)	0.322
Stroke or TIA, *n* (%)	0 (0.0)	1 (1.0)	1 (6 7)	0.001*

*A 2-sided p value <0.05 is considered of statistical significance

*AMI* acute myocardial infarction, *TIA* transient ischemic attack

^#^The analysis was restricted to patients who survived during the first month (*n* = 231)

^§^The analysis was restricted to patients who survived during the first 6-month (*n* = 228)

**Fig. 2:**
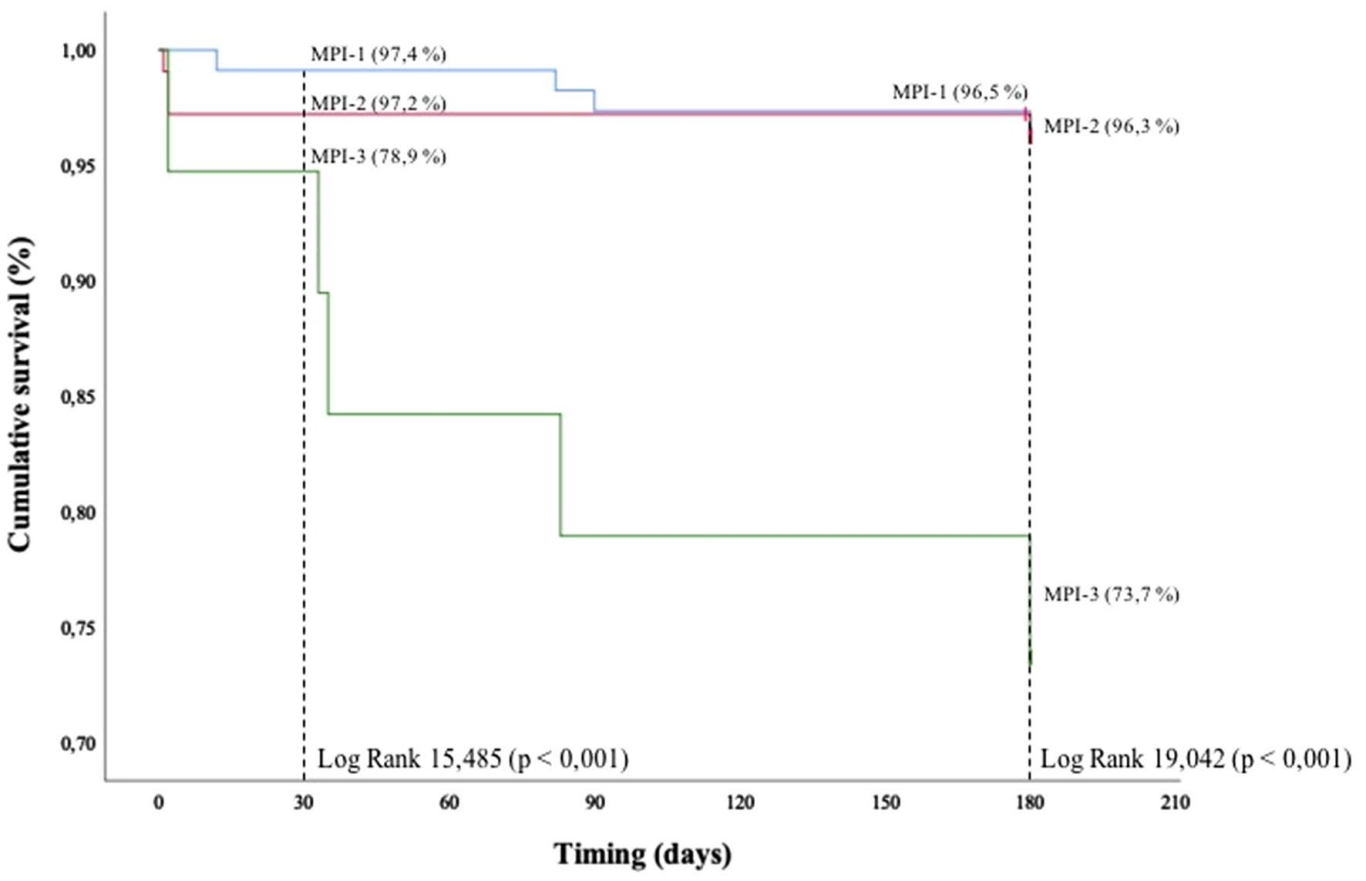
Estimated 30-day and 6-month mortality survival, using Kaplan–Meier analysis

The Kaplan–Meier analysis of the primary and secondary endpoints is shown in Fig. 2. Specifically, patients of MPI-3 had significantly high mortality rate regarding the primary endpoint with 30-day survival of 78.9%, compared to 97.4% and 97.2%, in MPI-1, MPI-2, respectively (LogRank 15,485; *p* < 0.001). The survival rate progressively decreased in the three MPI classes with a 6-month survival of 96.5%, 96.3%, 73.7% in groups MPI-1, MPI-2, and MPI-3 respectively (LogRank 19,042; *p* < 0.001).

## Discussion

Age is a major predictor of in-hospital and 6-month mortality in patients hospitalized for acute myocardial infarction, as well as independent predictor of PCI-related complications, including stent thrombosis [9–12]. Nevertheless, newer P2Y12 inhibitors that are able to reduce the risk of stent thrombosis are less prescribed in elderly [13], principally due to comorbidities and a higher risk of bleeding complications [13–15]. Moreover, frailty, per se, is also strongly associated with poor outcomes and high risk for mortality [16–19]. Particularly in the setting of acute myocardial infarction, the management of the elderly is very challenging due to lack of knowledge about the link between fragility, outcomes and interventional procedures. A detailed geriatric assessment may help to discriminate those requiring a more intensive treatment, more aggressive therapies and prolonged hospitalization. The MPI is a derived index based on six commonly used geriatric scales exploring cognitive, functional, nutritional and clinical status, as well as on information about drugs taken and patient’s social support [7]. Its short- and long-term predictive value has been widely established in the overall hospitalized population, and in older patients with pneumonia [20], dementia [21], heart failure [22] and transient ischemic attack [23]. Moreover, Pilotto et al. explored the role of MPI in predicting long-term all-cause mortality in older patients with chronic kidney disease [24]. The role of the CGA-based MPI stratification have been also assessed in older patients admitted to cardiology and internal medicine departments for cardiovascular diseases [25], as well as in older patients with coronary artery disease [26], or undergoing transcatheter aortic valve implantation for aortic stenosis [27, 28]. Even if focused on different aims, all these studies suggest that, in the context of “geriatric cardiology”, the multidimensional assessment of frailty as evaluated by the MPI [29], may be useful in taking clinical decisions in older patients with cardiac diseases. Similarly, our study demonstrates this concept in older patients with AMI who underwent PCI. In our “real-world” population of elderly hospitalized for AMI we observed poorer outcomes in patients belonged to higher MPI groups. The MPI evaluated at hospital admission was linearly and directly associated with length of in-hospital stay (particularly for hospitalization in cardiology ward) and in hospital adverse events, as cardiogenic shock, acute pulmonary oedema, new-onset of AF, need for definitive pacemaker implantation and infective complications. Additionally, the MPI evaluated at the moment of hospital admission, influenced significantly in-hospital mortality, 30-day and 6-month mortality. Specifically, during hospitalization no deaths occurred in MPI-1 group, while 2.8% and 10.5% of patients died for cardiovascular causes in MPI-2 and MPI-3 group, respectively. One month after AMI, mortality was significantly higher in MPI-3 with a rate of 21.1%, compared to 2.6% and 2.8% in MPI-1 and MPI-2, respectively. At 6-month follow-up the mortality rate was 26.3% for MPI-3, 3.5% for MPI-1 and 3.7% for MPI-2.

Moreover, it is noteworthy that in our study population the higher the MPI, the greater the risk of bleeding complications at 30-days. Interestingly, within the MPI-2 and MPI-3 groups, approximately 60% and 84% of patients were on clopidogrel treatment. Conversely, 65% of MPI-1 patients were on other P2Y12 platelet receptor inhibitors, especially ticagrelor. Although supported by a small sample size, the use of less potent antiplatelet agents in the more fragile group seems to correlate with a higher rate of stent thrombosis in the follow-up. These data confirm that new platelet inhibitor drugs are less prescribed in older patients. Additionally, a more use of oral anticoagulant therapy in MPI-3 group, in association with antiplatelet agents, could explain the high rate of bleedings among these patients. Our findings reflect that balancing the anti-ischemic benefits against the bleeding risk of antithrombotic agents is challenging in the management of the elderly with AMI. Therefore, a complete geriatric assessment may help to identify patients requiring more aggressive and prolonged antiplatelet regimens, as compared to those at higher risk of bleeding and so needing less intensive treatments.

We strongly believe that in the setting of AMI in older patients, an accurate multiparametric assessment is critical to guide a clinical decision process, which can influence patient prognosis. In older frail patients, with limited life expectancy and several comorbidities, inappropriate medical or interventional treatments should be avoided or managed with extraordinary care. Conversely, older patients with good clinical status and life expectancy should receive the standard level of care, including aggressive diagnostic and therapeutic interventions. A resulting clinical score may help cardiologists to stratify the patients and adapt the care, or recognize the cases where aggressive intervention may be futile.

### Limitations

Our study has some limitations, which should be considered when drawing conclusions. This is an observational single hospital study based on the multidimensional assessment of frailty, using the MPI, in a cohort of older patients with AMI treated with PCI, with a longitudinal follow-up design for all patients enrolled. The relatively small sample size, especially for the MPI-3 group, can limit the power of the study. Thus, the results should be confirmed and validated in a different and more representative population. Moreover, one-year follow-up results are not yet available in this paper, because it will be completed in October 2020. However, ongoing analysis of follow-up will complete the present findings. Finally, to substantiate the robustness of the results, studies with larger sample sizes and longer follow-up periods are required.

## Conclusion

In elderly patients suffering from AMI, frailty, assessed at hospital admission using a multiparametric test, is strongly associated with poor outcomes, including in-hospital mortality, 30-day and 6-month mortality, prolonged hospital care, and high risk of adverse events. The MPI offers a potential role for moving in clinical decision making for older adults with AMI, and it might change outcomes, increasing survival and reducing adverse events.

## Data Availability

The datasets generated and analysed during the study are available from the corresponding author on reasonable request.
